# Religiosity and Mental Health among Muslim Cancer Patients

**DOI:** 10.1192/j.eurpsy.2023.2004

**Published:** 2023-07-19

**Authors:** N. Becarevic, R. Softic

**Affiliations:** 1 Clinic of Psychiatry, University Clinic Center Tuzla, Tuzla, Bosnia and Herzegovina; 2 Clinic of Psychiatry, University Clinic Center Tuzla, Tuzla

## Abstract

**Introduction:**

Religiosity and spirituality are resources, frequently used by patients as a strategy against chronic diseases. Islam is the fastest growing monotheistic religion whose belief is based on the unity of God and devotion to God’s will, gratitude, and satisfaction with God’s provision. Despite many researches have proven mostly positive correlation between religion and health, there is a lack of them directed to a single religion.

**Objectives:**

To provide an overview of the most recent researches that have examined the role of Islam religion in cancer treatment.

**Methods:**

PubMed database was screened using the keywords, “Islam, religion, cancer, treatment”.

**Results:**

Patients expressed a lack of religiosity/spirituality support and it is connected to a significantly lower quality of life compared to those who adequately addressed their spiritual needs. The study which included 800 Muslim cancer patients showed that cancer patients (82.8%) prayed more than non-cancer individuals (72.5%). Many Muslim patients do not consider disease as a penance, but rather, as a redemption of sins, because they have trust and faith in God’s will. There are studies postulating the positive effects of fasting on cancer treatment.

**Conclusions:**

Muslim cancer patients are more religious and spiritual than the non-cancer Muslim population, and they are in need of spiritual support with the aim to reduce depression, anxiety, and stress. Health care professionals may encourage the patients to use their religious beliefs to cope with the challenges of therapy.

**Disclosure of Interest:**

None Declared

